# Post-exposure treatment of Ebola virus using passive immunotherapy: proposal for a new strategy

**DOI:** 10.1186/s40409-015-0003-1

**Published:** 2015-02-15

**Authors:** Jean-Philippe Chippaux, Leslie V Boyer, Alejandro Alagón

**Affiliations:** UMR 216, Mother and Child Facing Tropical Diseases, Research Institute for Development (IRD), Cotonou, Benin, and School of Pharmacy, Paris Descartes University, Sorbonne Paris Cité, Paris, France; Venom Immunochemistry, Pharmacology and Emergency Response (VIPER) Institute, University of Arizona, Tucson, Arizona USA; Institute of Biotechnology, National Autonomous University of Mexico (UNAM), Cuernavaca, Morelos Mexico; Institut de Recherche pour le Développement (IRD), 08 BP 841 Cotonou, Bénin

**Keywords:** Ebola, Epidemics, Immunotherapy, Prophylaxis, Africa

## Abstract

**Background:**

Better treatments are urgently needed for the management of Ebola virus epidemics in Equatorial Africa.

**Methods:**

We conducted a systematic review of the literature on the use of passive immunotherapy for the treatment or prevention of Ebola virus disease. We placed findings from this review into the context of passive immunotherapy currently used for venom-induced disease, and recent improvements in manufacturing of polyvalent antivenom products.

**Results:**

Passive immunotherapy appears to be one of the most promising specific treatments for Ebola. However, its potential has been incompletely evaluated, considering the overall experience and recent improvement of immunotherapy. Development and use of heterologous serum derivatives could protect people exposed to Ebola viruses with reasonable cost and logistics.

**Conclusion:**

Hyperimmune equine IgG fragments and purified polyclonal whole IgG deserve further consideration as treatment for exposure to the Ebola virus.

## Introduction

The epidemic of Ebola virus disease (EVD) currently taking place in West Africa has revived debate on the treatment of this severe infection [1]. Experimental treatment consisting of monoclonal antibodies has been used compassionately in half a dozen infected medical workers [2]. However, the efficacy and safety of these antibodies have not yet been evaluated in humans, and available quantities allow treatment of only a few patients. The small amount available and the anticipated high cost have precluded benefit for many patients, raising an additional serious problem of equity. During a WHO meeting in September 2014, experts agreed that developing treatments based on blood products from convalescent human patients was a priority [1]. However, heterologous animal products are not under clinical investigation.

Passive immunotherapy of animal origin has been used for over 120 years to treat bacterial and viral infections, envenomations and drug intoxications [3]. Its use decreased following the development of vaccination and antibiotics, with the notable exception of rabies, for which treatment with immune globulin remains frequent in Africa, Asia and Latin America [4,5]. Improvements in manufacturing through fragmentation and purification of immunoglobulin G (IgG), viral inactivation, lyophilization, and better understanding of pharmacokinetics have led to safer and more efficient products. Consequently, experience with millions of antivenom treatments annually offers a successful model now applicable to the treatment of EVD [6,7].

Passive immunotherapy of EVD has had insufficient consideration given to its conditions of use, benefits and limitations, including pharmacokinetics, potency, and dose. In the particular context of Ebola and the political, social and economic constraints of endemic countries, high technology may not be the most appropriate approach to disease management. Production of monoclonal antibodies is expensive and sometimes of low yield, significantly reducing the number of potential beneficiaries. Experience during EVD epidemics in Africa has involved insufficient resources and limited management capabilities by national health services. Under such circumstances, there are significant potential benefits in using equine polyclonal IgG fragments. Their manufacture is well standardized and inexpensive, compared to recombinant monoclonal antibodies. Moreover, their use can be managed directly by health personnel in at risk countries, enabling a more tailored and flexible response.

This study presents the arguments in favor of passive immunotherapy to control EVD after exposure, *i.e.* during either the incubation of the disease, or the disease itself. Polyclonal equine antibodies offer multiple potential benefits, including remarkable tolerance, availability, and ease of use. More importantly, such a treatment can be produced at a price affordable to the impoverished communities facing epidemic EVD.

## Review

### African Ebola virus and EVD

Ebola viruses belong to the family *Filoviridae*, which also includes *Marburgvirus* and *Cuevavirus* [8,9]. The incubation period ranges from 3 to 21 days and the illness lasts from 5 to 15 days. The disease starts abruptly with nonspecific symptoms that can be mistaken for other common diseases in Equatorial Africa such as malaria, yellow fever, typhoid or influenza [10]. Case fatality rates are very high although variable (between 20 and 80%) according to viral strain and possibly other factors such as the number of viral generations, mode of transmission, and availability of effective supportive care.

### History of passive immunotherapy of EVD

Behring and Kitasato [11] described passive immunotherapy, originally called “serum therapy” because it involved administration of whole serum, in 1890. Subsequently many diseases, including viral ones, benefited from serum therapy [3]. This gradually became “immunotherapy” as process improvements were introduced: precipitation of immunoglobulins, enzymatic digestion, and steps to reduce microbial contamination and purify the final product [6]. After the widespread introduction of antibiotics and immunization, however, heterologous immunotherapy was largely abandoned as an infectious disease treatment strategy [12]. Subsequently, the technology was advanced primarily for the purpose of neutralizing snake and scorpion venom.

Several therapeutic protocols for EVD have recently been suggested [13]. The first attempt to treat EVD with convalescent plasma was undertaken during the 1976 epidemics in Sudan and Democratic Republic of Congo (DRC). During these epidemics, a plasmapheresis program for obtaining convalescent plasma was implemented [14]. A Congolese patient with confirmed EVD received 500 mL of convalescent plasma (about 6 g IgG) and survived [10]. In addition, laboratory contamination occurred in Great Britain with samples from outbreaks. Six days after exposure (D6), clinical signs appeared in exposed people, corresponding to peak viremia. Interferon and symptomatic care were provided, without apparent improvement. On D8, 450 mL of convalescent plasma was administered to victims, with a second dose (330 mL) on D11 (*i.e.* nearly 10 g IgG). Clinical improvement occurred on D13, along with a significant decrease in viral load that disappeared on D15. Symptoms resolved on D18 and convalescence lasted 10 weeks [15]. It was not possible to draw firm conclusions from these two cases, especially since the second patient recovered within a period compatible with a natural recovery.

During the 1995 outbreak in Kikwit (DRC), eight patients received transfusions of convalescent human plasma, ranging from 150 to 450 mL (1.5 to 5 g IgG), 4–15 days after the onset of clinical signs, and seven survived [16]. Again, results were not considered conclusive, because of small sample size and variable timing.

Dye *et al*. [17] treated three monkeys during the clinical phase of the disease using polyclonal IgG from monkeys that had survived an infection with Ebola virus in controlled conditions. The monkeys showed minimal illness, followed by full recovery.

Goats and horses were hyperimmunized with the culture medium and extracts of monkey liver infected with *Zaire ebolavirus* [18]. Purified IgG protected experimentally infected guinea pigs and baboons. In addition, goat hyperimmune IgG was given to four persons who were accidentally exposed to infectious laboratory materials, without any confirmation of contamination. Horse IgG was also evaluated independently in a *Macaca cynomolgus* model. In these monkeys, viremia and clinical signs appeared later than in controls showing a reduced replication of the virus but not complete stop, despite use of interferon with passive immunotherapy [19].

During the Kikwit outbreak, human monoclonal antibodies were constructed according to the techniques of “phage display” from two patients’ bone marrow RNA [20]. These antibodies react with the nucleoprotein, envelope glycoprotein and non-structural secretory glycoprotein secreted by infected cells. It was observed that neutralizing antibodies are produced at a relatively low yield during infection, which could partly explain the failure of some treatments using convalescents’ plasma [21].

A mixture of two chimeric monoclonal antibodies (ch133 and ch226) against *Zaire ebolavirus* was effective in rodents, but protected only one out of three infected rhesus monkeys [22]. Monoclonal antibodies were intravenously administered (50 mg per animal), 24 and 72 hours after viral challenge. Monoclonal antibodies remained detectable in the blood of surviving animals until the appearance of antibodies induced by the infection. In contrast, the serum concentration of monoclonal antibodies became undetectable at the terminal stage of the disease in the two monkeys that died due to the infection whereas viremia increased inversely. This suggests that the virus consumes large amounts of neutralizing antibodies.

A combination of three neutralizing monoclonal antibodies (1H3, 2G4, and 4G7) directed against two glycoproteins and one secretory glycoprotein of Ebola virus was used in a *Macacus cynomolgus* model [23]. Four infected monkeys survived following treatment with the monoclonal antibody administered (25 mg · kg^−1^) three consecutive days starting 24 hours after the viral challenge. The same treatment, starting 48 hours after infection, resulted in two deaths out of four macaques, suggesting late treatment is less effective because either the pathogenic effects are more developed or the dose was insufficient compared to the amount of virions.

Three chimeric humanized monoclonal antibodies against Ebola virus (c13C6, h-13 F6, and c6D8; their combination is known as MB-003) were produced by an ovarian cell line of the Chinese hamster (*Cricetulus griseus*) and a whole plant, *Nicotiana benthamiana* [2]. The antibodies of these two systems, respectively at doses of 50 mg · kg^−1^ and 16.7 mg · kg^−1^, protected rhesus monkeys from lethal viral challenge when administered one hour prior to treatment. Protection was significant when monoclonal antibodies were administered 24 and 48 hours after infection. In all experiments, surviving animals showed no viremia and few or no clinical symptoms [2]. This monoclonal antibody cocktail, composed of one component from the MB-003 mixture and two from ZMab (consisting of murine mAbs m1H3, m2G4 and m4G7), has been used recently in some patients from the West African epidemic, under the name ZMapp^™^. In another study with nonhuman primates, three doses of 50 mg · kg^−1^ of an optimized combination of ZMapp^™^ have been administered every three days, resulting in the recovery of six monkeys infected with 2,512 PFU three days before the first injection of ZMapp^™^, during which time they developed viremia and signs of infection [24].

### Strategy for passive immunotherapy and prophylaxis of EVD

Post-exposure treatment of EVD may well be compared to the post-exposure treatment of other viral diseases such as rabies. However, there are two main differences. First, the Ebola virus spreads in the body much faster than the rabies virus. In the post-exposure situation, this increases the relative importance of rapid administration of passive immunotherapy as opposed to active immunization with a vaccine that will not have time to act [25]. Second, unlike the rabies virus, Ebola virus is not sequestered in the nervous system where antibodies cannot penetrate, which has advantageous implications both for the site of injection and for the bioavailability of neutralizing antibodies [25].

Convalescent human plasma, recently reconsidered, is difficult and dangerous to collect and administer [1]. *Filovirus* persist several weeks in the body after recovery [26]. In addition, antibody titers appear to be too low for good protection. Obtaining antibodies from another source remains crucial.

Production and use of animal-derived immunotherapeutic agents is a complex process dependent on many factors. The quality and specificity of immunogens, host species, and individual immune response determine the neutralization titer, and consequently the potency and efficacy of the preparation [6,7]. Enzymatic digestion of IgG, purification and elimination of potentially infectious agents improve the safety of the product [27]. The therapeutic dose depends not on the patient’s body weight, but on the amount of antigen present in the body and its tissue distribution. The distribution of antibodies is related to their composition (whole IgG or IgG fragments) and their route of administration [28,29]. IgG and F(ab’)_2_ remain in the compartment where they are introduced, whereas smaller fragments including Fab or Fv have a greater volume of distribution. Sufficient blood concentration of antibodies enables the gradual transfer of antigens from the peripheral compartments towards the blood where they are bound and cleared [28,30]. In addition, the half-life of IgG and F(ab’)_2_ is 5 to 10 times longer than that of the Fab or Fv, which require more frequent dosing [28].

Implementation of passive immunotherapy can be divided into seven steps:

1. Antigen production

The use of culture medium or extracts from Ebola-infected animal organs does not seem a promising strategy since the process involves substantial risks [18]. Therefore, it is preferable to produce isolated virus proteins–possibly recombinant– or proteins vectorized by a non-pathogenic virus.

The selection of immunogens is logically directed to proteins involved in the infectious capacity of Ebola virus, such as the glycoproteins, or those that have high pathogenicity, such as VP24 and VP35 viral proteins. The main limitations of this approach involve the specificity of these proteins and the ability of the antibodies produced against them to neutralize viruses belonging to other species. It may be preferable to have polyvalent antibodies, as in the case of antivenoms [6,7]. Among species of Ebola virus pathogenic to humans, the homology of amino acid composition is 55 to 70% for the glycoproteins and VP24 or VP35 viral proteins, which requires measuring the level of paraspecificity among the different species of Ebola virus or identifying conserved structures involved in immunogenicity [31].

Immunogens may be recombinant proteins or expressed by a recombinant vesicular stomatitis virus (rVSV), or other recombinant virus, carrying Ebola virus antigens. Immunization with recombinant rVSV generated cross-immunity in different species of Ebola virus, although such immunization should ideally involve a cocktail of antigens [32-34]. The rVSV models appear to be effective and harmless to the host [35].

In addition, immunogens should be capable of inducing a good immune response both quantitatively and qualitatively. Five epitopes involving monoclonal antibodies protective against Ebola virus in the murine model have been identified in the glycoprotein of Ebola virus, one of which is conserved in all known Ebola viruses [36]. These genetically engineered proteins have shown high immunogenicity [37]. However, Ito *et al*. [38] showed that the glycoprotein can inhibit the secretory specific neutralizing antibodies against the envelope glycoprotein. It appeared that the epitopes of both proteins corresponding to neutralizing antibodies were similar and that the secretory glycoprotein served as decoy for neutralizing antibodies. The study of the mechanisms of neutralization by monoclonal antibodies showed that they inhibited viral transduction by two different routes [39]. Neutralization by one of them (KZ52) inhibits the endosomal proteolytic activity necessary for Ebola virus penetration into the cell. Another monoclonal antibody (JP3K11) inhibits the melting and/or binding of the membrane receptor by the structural glycoprotein. According to the authors, antibodies that recognize the reduced post-glycoprotein, as JP3K11, could be more effective.

Furthermore, Bale *et al.* [40] showed that access of the antibody against the corresponding epitope and the affinity for the antigen were important to explain the neutralizing capacity of the antibody.

2. Animal immunization

The horse is the animal most used in production of hyperimmune sera because of the ease of management, high antibody yield and low risk of human contamination by virus or unconventional infective agents. Immunization itself is standardized and performed under optimal conditions for both personnel and animals [6,7]. With optimal animal husbandry, titers of neutralizing antibodies produced in hyperimmunized horses may exceed those expected from simple immunization, by a factor of 10- to 20-fold (A. Alagón, unpublished data).

3. Immunological tests

Although increased antibody titers in immunized animals are assessed by ELISA, neutralizing capacity must be determined in an appropriate animal model (rodents or primates) or cell culture using rVSV carrying Ebola virus proteins [41].

It is likely that the neutralization titer and/or the dose of antibodies used in experiments conducted to date have been insufficient. In some of them, inappropriate doses or administration routes may have not resulted in a sufficient plasma concentration of antibodies to neutralize the virus during the course of the disease. Similarly, long intervals between serial doses of monoclonal antibodies could result in ineffective sustained levels to prevent virus replication, limiting the effect of passive immunotherapy. Both phenomena have been observed in treatment of snakebite envenomation, in which repeated doses of antivenom are sometimes required to overcome pharmacokinetic-toxicokinetic mismatch. There is, however, a dramatic difference in the expected amount of antibodies between viral infection and envenomation. In the latter, the amount of venom injected and final antigen concentrations in the body decrease with natural clearance of the venom and, optionally, neutralization by antivenom [6]. In contrast, during a viral infection, the virus replicates and the amount of antigen increases exponentially. Consequently, the concentration of antibodies in the body must be very high to neutralize viral antigens, particularly if treatment is initiated late in the course of disease. Dose and frequency of antibody administration must take such factors into consideration, meaning that treatment of fulminant Ebola could involve larger quantities of antibodies than previously administered.

4. Fragmentation and purification of IgG

Some manufacturers are specialized in production of heterologous antibody fragments at a reasonable cost. Production is now standardized and risks resulting from the use of well-purified IgG fragments are trivial compared to the historical experience with whole IgG [7]. Currently, it is possible to administer more than 500 mg of antibody or even several grams, with a low incidence of adverse events, most of them mild [27]. The incidence of side effects from equine antivenom administration has been reduced from 80% (with nearly 25% of anaphylactic shock) with whole IgG preparations, to less than 10% of mild reactions with highly purified IgG fragments [27]. Highly purified equine IgG fragment preparations are now used safely for treatment of snakebite in Africa [7].

5. Formulation and dosage of passive immunotherapy

The choice of whole IgG, Fab or F(ab’)_2_ fragment should consider the distribution in the different compartments of the body, and their respective half-life as well as mechanisms of action. F(ab’)_2_ remains longer than Fab in the blood compartment, where it binds with antigens rapidly cleared by the reticuloendothelial system [28,30]. Furthermore, F(ab’)_2_ remains five to six times longer in the body than Fab, thereby reducing the frequency of required dosing. Moreover, although Fab may form a complex with antigen in any body compartment, it must return to the plasma compartment for clearance; this may be problematic, depending on the size of the immune complex. Whole IgG offers the potential advantage of complement-dependent cytotoxicity (CDC) and antibody-dependent cellular cytotoxicity (ADCC) in addition to the steric hindrance afforded by Fab and F(ab’)_2_ fragments, with a necessary trade-off in characteristics that may warrant comparative *in vivo* studies. Fab and F(ab’)_2_ fragments cannot mediate antibody-dependent enhancement of infection with Ebola virus, which involves interaction between whole IgG and the Fc receptor or complement C1q and the C1q receptor [42].

The pharmacokinetics of whole IgG or its fragments are not determined by the epitopes of antigen recognition. Intravenous administration of IgG fragments, whatever their composition, guarantees rapid delivery, with a bioavailability close to 100%, either in the blood compartment for F(ab’)_2_, or in all the compartments for Fab [28,30]. Intramuscular administration of F(ab’)_2_ results in a slow and partial (about 50%) spreading of the inoculum from the injection site. This is useful if a gradual release of antibodies is desired (e.g. in anticancer drugs), rather than immediate, as for the treatment of envenomation or active infections [29].

Dose should be based on total antigen load, in this case viremia, rather than body weight or volume. During envenomation, antigen load may be estimated statistically (relative to the average venom yield of the snake) or based on the rapidity of onset and severity of clinical symptoms. Typically, one vial of antivenom contains 200 mg of antivenom F(ab’)_2_ to be diluted in 10 mL of solvent, which corresponds to approximately 300 mg of IgG. However, it is possible to increase the protein dose of each vial and to adjust the dilution and rate of administration to limit the occurrence of undesirable effects.

6. Clinical trials

For forty years, therapeutic use of purified IgG fragments has been well established [7]. Manufacturing practice must be sufficiently well controlled to allow use in humans. However, the specificity of antibodies with respect to antigenic epitopes does not affect their safety and tolerability and, therefore, these are not limiting factors in new product development. Accordingly, as in the case of antivenoms, phase I studies are not required.

Phase II trials of candidate products in patients or persons who have been in contact with EVD cases could be ethically permitted, under proper conditions and new WHO recommendations [1]. In compassionate treatment of a highly lethal disease, and subject to the opinion of an ethics committee and administrative authorization by appropriate health authorities, it would be possible to treat many patients relatively quickly after preclinical testing. As in the case of antivenom, concurrent controls would not be necessary, unless there is another treatment for comparison [43]. In the event of promising animal data, in which use of a placebo would be unethical, clinical improvement and reduction of viremia could be measured against historical expectations. Finally, pragmatic clinical trials – not explanatory – may be more appropriate [44].

7. Distribution and storage of F(ab’)_2_

Lyophilized F(ab’)_2_ products have an estimated room temperature shelf life of 3 to 5 years depending on the pharmacopoeia policy, but they actually retain potency much longer [6]. These could be stored in each endemic country, ready to use on short notice in the event of an epidemic. Current production capacity exceeds several hundred thousand doses annually, which means that a practical scale-up at existing facilities could provide an adequate supply.

Approximate cost can be anticipated, based on the current production and sale of antivenoms in Africa. Following initial development, 600 milligrams of specific F(ab’)_2_ (corresponding to approximately 1 g of specific IgG) would cost about US$ 65–80, in contrast with the usual cost of monoclonal antibodies [45].

## Conclusion

Hard to control due to the current health and socioeconomic contexts, EVD outbreaks represent an emerging and growing public health problem in sub-Saharan Africa. The delay in development of an effective and accessible therapy shows the inability of the international community to mobilize effectively, despite an unprecedented scale. Passive immunotherapy, although tested in early outbreaks in Sudan and DRC, has still not been fully evaluated. Long experience with the use of antivenom could be used to reformulate the conditions and procedures for the use of highly purified equine specific IgG fragments to treat persons exposed to the Ebola virus or affected by EVD. A treatment protocol inspired by the existing standard for post-exposure management of rabies could be applied, taking into account the distinct characteristics of EVD, such as the speed of viral replication and the presence of virus in compartments accessible to IgG and IgG fragments.

If an F(ab’)_2_ proves to be effective, the implementation of a strategy for controlling epidemics by passive immunotherapy will be accessible, relatively inexpensive, and easily applicable.
